# PRITrans: A Transformer-Based Approach for the Prediction of the Effects of Missense Mutation on Protein–RNA Interactions

**DOI:** 10.3390/ijms252212348

**Published:** 2024-11-17

**Authors:** Fang Ge, Cui-Feng Li, Chao-Ming Zhang, Ming Zhang, Dong-Jun Yu

**Affiliations:** 1 State Key Laboratory of Organic Electronics and Information Displays & Institute of Advanced Materials (IAM), Nanjing University of Posts & Telecommunications, 9 Wenyuan, Nanjing 210023, China; gfang0616@njupt.edu.cn; 2 School of Computer, Jiangsu University of Science and Technology, 666 Changhui Road, Zhenjiang 212100, China; 231210701110@stu.just.edu.cn (C.-F.L.); 221210701126@stu.just.edu.cn (C.-M.Z.); zhangming@just.edu.cn (M.Z.); 3 School of Computer Science and Engineering, Nanjing University of Science and Technology, 200 Xiaolingwei, Nanjing 210094, China

**Keywords:** missense mutation, protein-RNA interactions, binding affinity, protein language model, bioinformatics

## Abstract

Protein–RNA interactions are essential to many cellular functions, and missense mutations in RNA-binding proteins can disrupt these interactions, often leading to disease. To address this, we developed PRITrans, a specialized computational method aimed at predicting the effects of missense mutations on protein–RNA interactions, which is vital for understanding disease mechanisms and advancing molecular biology research. PRITrans is a novel deep learning model designed to predict the effects of missense mutations on protein–RNA interactions, which employs a Transformer architecture enhanced with multiscale convolution modules for comprehensive feature extraction. Its primary innovation lies in integrating protein language model embeddings with a deep feature fusion strategy, effectively handling high-dimensional feature representations. By utilizing multi-layer self-attention mechanisms, PRITrans captures nuanced, high-level sequence information, while multiscale convolutions extract features across various depths, thereby enhancing predictive accuracy. Consequently, this architecture enables significant improvements in ΔΔG prediction compared to traditional approaches. We validated PRITrans using three different cross-validation strategies on two newly reconstructed mutation datasets, S315 and S630 (containing 315 forward and 315 reverse mutations). The results consistently demonstrated PRITrans’s strong performance on both datasets. PRITrans demonstrated strong predictive capability, achieving a Pearson correlation coefficient of 0.741 and a root mean square error (RMSE) of 1.168 kcal/mol on the S630 dataset. Moreover, its robust performance extended to independent test sets, achieving a Pearson correlation of 0.699 and an RMSE of 1.592 kcal/mol. These results underscore PRITrans’s potential as a powerful tool for protein-RNA interaction studies. Moreover, when tested against existing prediction methods on an independent dataset, PRITrans showed improved predictive accuracy and robustness.

## 1. Introduction

Protein-RNA interactions are fundamental to various cellular processes, including post-transcriptional regulation [1] and protein synthesis [2,3,4]. Mutations have been identified in over 1000 RNA-binding proteins (RBPs) implicated in human diseases, representing more than 20% of proteins with documented mutations [5]. These mutations can significantly impact the properties, expression levels, and interaction networks of RBPs, thereby altering their regulatory functions on downstream RNA targets. Moreover, accumulating evidence indicates a strong association between RBPs with tumor initiation and progression. In cancer cells, RBPs regulate target gene expression, influencing critical biological processes such as malignant proliferation, transformation, migration, and invasion [6]. For example, in vertebrates, the Y-box binding protein-1 is highly expressed in multiple cancer types, including breast, lung, colon, and gastric cancers [7], and exerts oncogenic effects by modulating various stages of gene expression [8,9,10,11]. Missense mutations in RNA-binding proteins can disrupt these interactions, leading to alterations in free energy. These disruptions may significantly disrupt or abolish the normal functions of these proteins, potentially causing diseases like cancer and severe combined immunodeficiency [12,13,14]. Thus, understanding mutation effects on protein–RNA interactions is vital for understanding disease mechanisms and developing specific treatments [15].

Traditional techniques (such as isothermal titration calorimetry [16], surface plasmon resonance [17], and fluorescence resonance energy transfer [18]) have been utilized to determine the binding free energy between proteins and RNA. Despite their accuracy, these methods are both expensive and labor-intensive, making them impractical for the high-throughput analysis needed for expanding genomic datasets [15]. This underscores the critical need for computational models capable of predicting binding affinity changes. Such approaches could significantly advance large-scale assessments of how missense mutations affect protein-RNA interactions, thus supporting the identification and analysis of disease-relevant mutations.

Despite considerable advancements in computational methods for predicting and modeling the effects of missense mutations on protein stability [19,20,21,22,23,24] and protein–protein interactions [25,26,27,28], accurately predicting their impact on protein–RNA/DNA interactions remains particularly challenging. This complexity arises primarily from the nature of nucleic acid chemistry and binding, which limits the availability of high-quality experimental data [29]. Additionally, prior studies have highlighted significant differences between protein–DNA and protein–RNA interactions [15,30]. Consequently, only a limited number of computational methods have been proposed to address this challenge. To date, Pires et al. have proposed mCSM-NA, which predicts changes in binding affinity for protein–DNA/RNA interactions by combining graph-based signatures and pharmacophore features [31]. Similarly, Peng et al. developed SAMPDI, a linear regression model that predicts changes in protein–DNA binding affinity after a single mutation by modifying molecular mechanics/Poisson–Boltzmann surface area energy terms and incorporating knowledge-based descriptors [32]. Furthermore, Zhang et al. introduced PremPDI, which employs molecular mechanics force fields and statistical potential methods to estimate the impact of missense mutations on protein-DNA interactions [33]. Building on this, they later developed PremPRI, which uses a combination of three sequence and eight structural features of several linear regression models for predicting the effects of single mutations in RNA-binding proteins [29]. Additionally, Yao et al. conducted a systematic comparison of missense mutations in DNA- and RNA-binding proteins. Their findings revealed that these mutations could exhibit similar or distinct trends in binding free energy changes, depending on the nature of the mutated residues [15]. They further developed PEMPDI, which integrates novel geometric partition energy features and interface structural characteristics to predict mutations in DNA- and RNA-binding proteins [15]. Additionally, several classification-based methods have been developed to predict hotspots at protein–RNA binding interfaces [34,35,36].

In conclusion, despite advancements made by machine learning or deep learning approaches in predicting protein–RNA interactions, they still face limitations in broad applicability, particularly in accurately predicting high ΔΔG values resulting from mutations. Deep learning models, known for their rapid analysis and predictive accuracy, have excelled in action classification, speech recognition, and natural language processing [37,38,39,40,41]. Notably, the Transformer framework has proven effective in predicting the effects of single mutations on protein stability [42]. Advanced protein language models, like ESM-2 [43] and ProtTrans [44], further improve prediction accuracy by capturing intricate patterns within protein sequences. Thus, integrating deep learning models, the Transformer framework, and embeddings from ESM-2 and ProtTrans holds significant promise for enhancing the prediction of missense mutation impacts on protein–RNA interactions. In this study, we present PRITrans, a novel approach designed to increase the precision of these predictions. PRITrans combines amino acid-level embeddings from ESM-2 [43] and ProtTrans [44] with Transformer and multiscale convolutional modules to deliver accurate and efficient predictions. PRITrans addresses existing challenges by utilizing an advanced network architecture and comprehensive feature representations, effectively managing high-dimensional data and excelling in predicting high ΔΔG mutations. Our validation on benchmark datasets using three cross-validation strategies shows that PRITrans outperforms existing methods on independent test sets. The newly reconstructed datasets and source code for PRITrans are freely available at https://github.com/cuifengLI/PRITrans (accessed on 14 November 2024).

## 2. Results and Discussion

### 2.1. Model Evaluation with Three Cross-Validation Strategies on Forwardand Reverse Mutations

**Figure 1:**
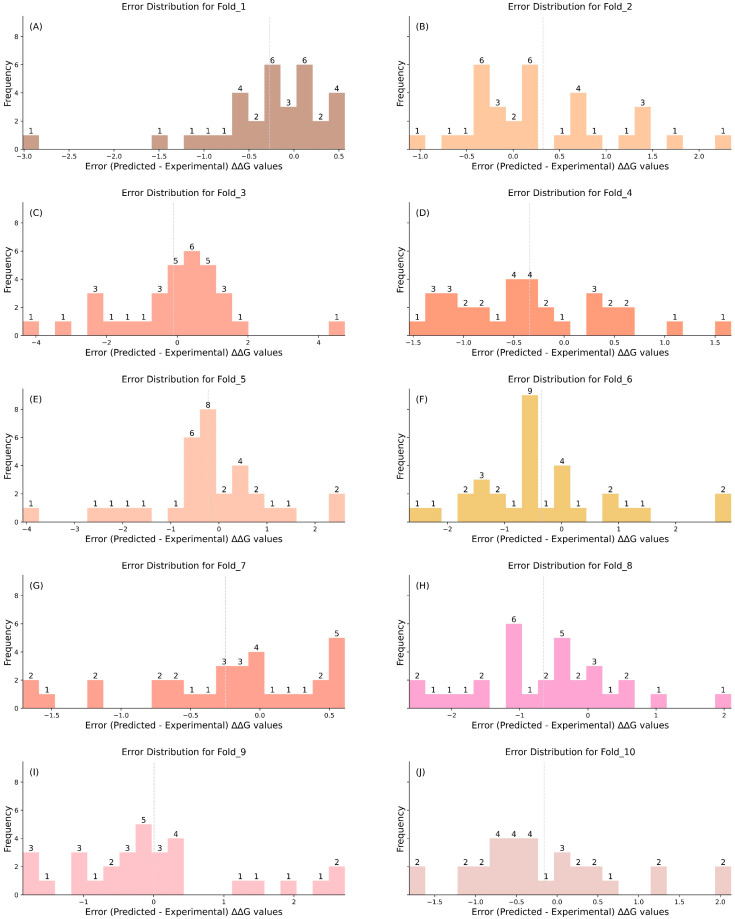
Error distribution for each fold in the S315 dataset using CV3. (**A**–**J**) depict the error (predicted–experimental) ∆∆G value distributions for Fold_1 to Fold_10. Note: the dotted lines in each histogram denote the mean error per fold, highlighting the central tendency and potential biases in the error distribution.

**Figure 2:**
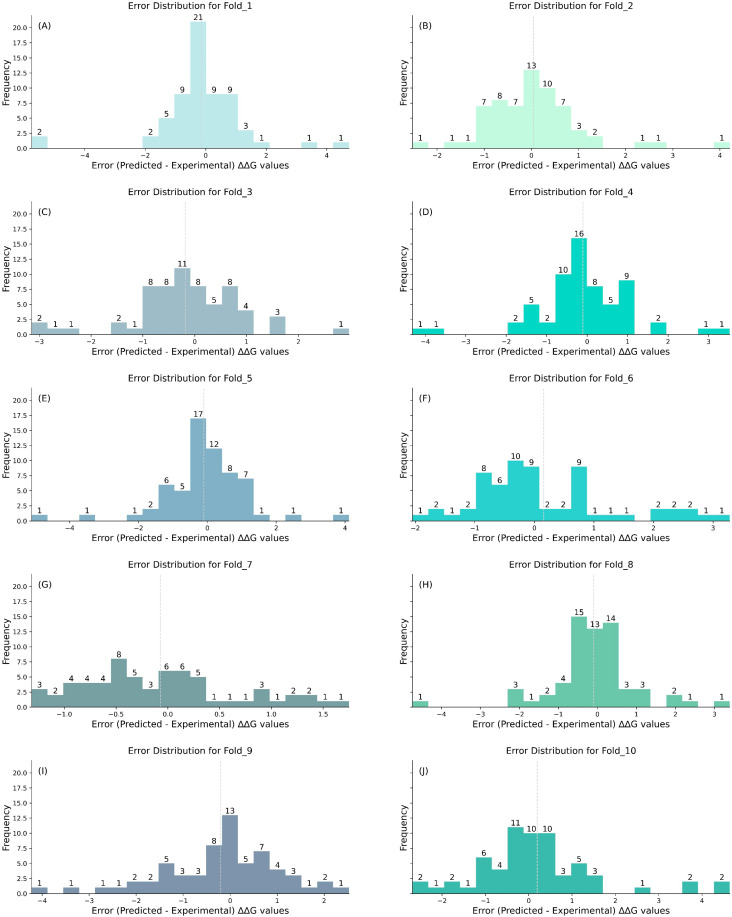
Error distribution for each fold in the S630 dataset using CV3. (**A**–**J**) depict the error (predicted–experimental) ∆∆G value distributions for Fold_1 to Fold_10. Note: the dotted lines in the histograms indicate the mean error for each fold, serving as a visual marker for the central tendency of the error distribution.

Table 1 presents PRITrans’s performance on the S315 dataset using three cross-validation strategies (CV1, CV2, and CV3), while Figure S2 visualizes these results. To address earlier limitations, we expanded S315 by incorporating reverse mutations, creating the S630 dataset. The same cross-validation strategies were applied to S630, with the results displayed in Table 1 and Figures S1–S3. Figure 1 and Figure 2 depict the error distributions for each fold under CV3.

(1) CV1, CV2, and CV3 comparison on S315

As shown in Table 1 and Figure S1, PRITrans yielded a mean PCC of 0.776 ± 0.048, RMSE of 0.768 ± 0.094 kcal/mol, and MAE of 0.557 ± 0.052 kcal/mol across 20 repetitions of CV1. For CV2, the average PCC was 0.743 ± 0.034, RMSE was 0.744 ± 0.054 kcal/mol, and MAE was 0.538 ± 0.039 kcal/mol. Although CV2’s PCC was 0.033 lower, it showed marginally better RMSE and MAE performance. CV2’s smaller PCC standard deviation (0.034 vs. 0.048 in CV1) also indicates greater model stability. These results suggest that CV2, despite using less training data (80% vs. 90% in CV1), delivered similar or slightly better performance due to a more balanced data split, improving generalization. In CV3, the median PCC dropped to 0.61, RMSE rose to 1.07 kcal/mol, and MAE increased to 0.83 kcal/mol. CV3 exhibited greater variability, with PCC from 0.31 to 0.83 and RMSE from 0.70 to 1.63 kcal/mol, reflecting reduced accuracy and stability, likely due to dataset limitations and uneven data splits. The small proportion of mutations with ΔΔG < 0 (31 out of 315) further contributed to these inconsistencies.

(2) CV1, CV2, and CV3 comparison on S630

As demonstrated in Table 1 and Figures S2 and S3, the average performance metrics for CV1 over 20 repetitions showed a PCC of 0.729 ± 0.047, RMSE of 1.250 ± 0.094 kcal/mol, and MAE of 0.794 ± 0.060 kcal/mol. CV2, while yielding a slightly lower PCC of 0.728 ± 0.017, exhibited improved RMSE at 1.197 ± 0.038 kcal/mol and a marginally higher MAE at 0.850 ± 0.033 kcal/mol. The heatmaps in Figure S2 further underscore this trend, with CV2 consistently outperforming CV1 in most repetitions, highlighting enhanced model stability and better prediction accuracy in terms of error minimization. CV3 displayed a PCC of 0.741 ± 0.065, RMSE of 1.168 ± 0.205 kcal/mol, and MAE of 0.809 ± 0.112 kcal/mol. While CV3 showed a slight improvement in PCC over both CV1 and CV2, the RMSE and MAE remained within comparable ranges. This suggests that although CV3 may achieve higher PCC, its accuracy in minimizing prediction errors does not significantly differ from the other CV strategies. These variations across CV1, CV2, and CV3 can likely be attributed to differences in data partitioning and randomization effects during cross-validation, which influence model generalization.

(3) Error distribution analysis of CV3 results on S315 and S630

Figure 1 and Figure 2 illustrate the error distributions of CV3 on the S315 and S630 datasets. This comparison provides a thorough assessment of PRITrans performance and reveals key differences in the error patterns between the two datasets.

For S630, the prediction errors are tightly concentrated between −2 and 2, with most values near zero, indicating improved precision with the expanded dataset. In contrast, while S315 errors also fall within this range, their distribution is more dispersed, particularly in certain folds, reflecting broader variations.

Detailed Fold Comparison. Fold_1 to Fold_5: (i) S630 dataset: errors remain centralized between −2 and 2, demonstrating higher precision. (ii) S315 dataset: although within the same range, the errors are more scattered, especially in Folds 3 and 5. Fold_6 to Fold_10: (i) S630 dataset: errors continue to be tightly clustered, with minimal deviation in Folds 7 and 8. (ii) S315 dataset: the error range broadens, extending from –4 to 4, particularly in Folds 9 and 10, suggesting higher variability and reduced prediction stability.

Error Standard Deviation: As shown in Table 1, the standard deviation of the 10-fold PCC for S630 is 0.065, with RMSE and MAE deviations of 0.205 and 0.112, respectively. In contrast, S315 exhibits higher variability, with a PCC deviation of 0.184, and RMSE and MAE deviations of 0.307 and 0.226. These results indicate that the expanded S630 dataset enhances the stability and predictive performance of the PRITrans model, particularly in handling both forward and reverse mutations.

### 2.2. Comparative Analysis of Prediction Performance with Various Modules

#### 2.2.1. Impact of the Encoder Module on PRITrans Performance

To assess the contribution of the encoder module in PRITrans, we conducted four ablation experiments. Experiment 1: dimensionality-reduced ESM-2 and PT embeddings were concatenated and passed through a fully connected layer (ESM-2_p_ and PT_p_). Experiment 2: ESM-2 embeddings were processed through the encoder module, while PT embeddings remained as in Experiment 1 (ESM-2_Ep_ and PT_p_). Experiment 3: PT embeddings were processed through the encoder module, while ESM-2 embeddings were treated as in Experiment 1 (ESM-2_p_ and PT_Ep_). Experiment 4: both ESM-2 and PT embeddings were processed through the encoder module (ESM-2_Ep_ and PT_Ep_). All experiments used identical hyperparameters and were evaluated with CV3 on the S630 dataset, encompassing forward and reverse mutation data. Results are summarized in Table 2.

The results in Table 2 highlight the effectiveness of the encoder module. In Experiment 2, applying the encoder to ESM-2 embeddings increased PCC to 0.641, while reducing the RMSE and MAE to 1.339 and 0.943, respectively, outperforming Experiment 3. Experiment 4, where the encoder was applied to both embedding types, achieved the best performance with a PCC of 0.670, RMSE of 1.315, and MAE of 0.918. These results demonstrate that applying the encoder to ESM-2 embeddings yields benefits, with the most substantial gains observed when applied to both ESM-2 and PT embeddings simultaneously.

#### 2.2.2. Contribution of the Multiscale Convolution Module to Performance

We evaluated the impact of the multiscale convolution module through the following experiments. Experiment 1: ESM-2 embeddings processed with the multiscale convolution module, referred to as ESM-2_m_, with PT embeddings untreated (PT_p_). Experiment 2: PT embeddings processed with the multiscale convolution module (PT_m_), while ESM-2 embeddings remained untreated (ESM-2_p_). Experiment 3: both ESM-2 and PT embeddings processed with the multiscale convolution module, denoted as ESM-2m and PTm. All experiments used identical hyperparameters and were assessed using CV3 on the S630 dataset. Results are presented in Table 3.

Table 3 indicates that the multiscale convolution module improves performance when applied to PT embeddings, with an increase in PCC of 0.074 and reductions in RMSE and MAE by 0.088 and 0.081, respectively. However, its application to ESM-2 embeddings alone resulted in diminished performance. The best outcome was achieved by applying the module to both embedding types, yielding a PCC of 0.750, RMSE of 1.207, and MAE of 0.861.

#### 2.2.3. Synergistic Effects of Combining Encoder and Multiscale Convolution Modules

Building on the findings from Section 2.2.1 and Section 2.2.2, we further examined the impact of combining the encoder and multiscale convolution modules. Experiment 1: ESM-2 embeddings processed through the encoder module (ESM-2_Ep_). Experiment 2: PT embeddings processed through the multiscale convolution module (PT_m_). Experiment 3: outputs from ESM-2_Ep_ and PT_m_ concatenated for final prediction. All experiments used consistent hyperparameters and were evaluated with CV3 on S630. The results are detailed in Table 4.

Table 4 reveals that integrating both modules (ESM-2_Ep_ and PT_m_) led to significant performance gains, with a 0.067 and 0.14 increase in PCC compared to using ESM-2_Ep_ or PT_m_ alone. Additionally, RMSE and MAE were further reduced, indicating improved accuracy and stability. These results underscore the advantage of using specialized processing for each embedding type followed by their integration, resulting in notable enhancements to the PRITrans model’s predictive performance.

### 2.3. Comparison with Existing Methods

**Figure 3:**
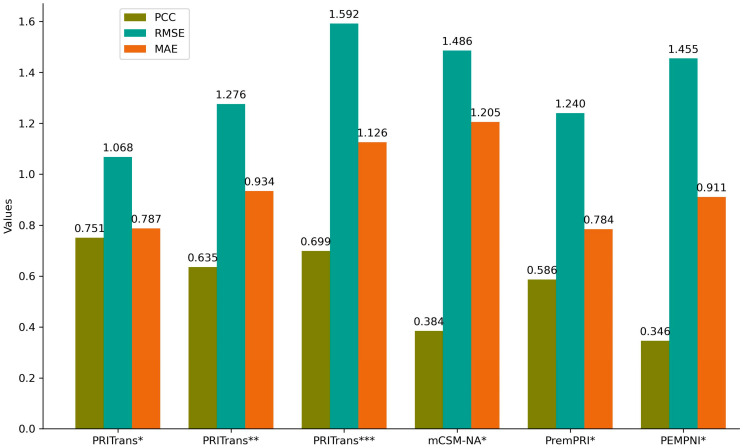
Performance comparison of PRITrans and existing predictors using S79 mutation data. Note: PRITrans*, trained on forward data using CV3. PRITrans**, trained on the entire dataset using CV3. PRITrans***, trained on the entire dataset using CV3 and evaluated on the S158 dataset, including reverse mutations. mCSM-NA*, excludes the 15 mutation data points with the highest squared errors between predictions and experimental ΔΔG values. PremPRI*, missing predictions for PDB_IDs 1C9S (10), 4MDX (2), and 5EV1 (1) were substituted with experimental ΔΔG values. PEMPNI*, missing predictions for PDB_IDs 1VS5 (2), 3OL6 (1), and 5W1H (1) were replaced with experimental ΔΔG values.

**Figure 4:**
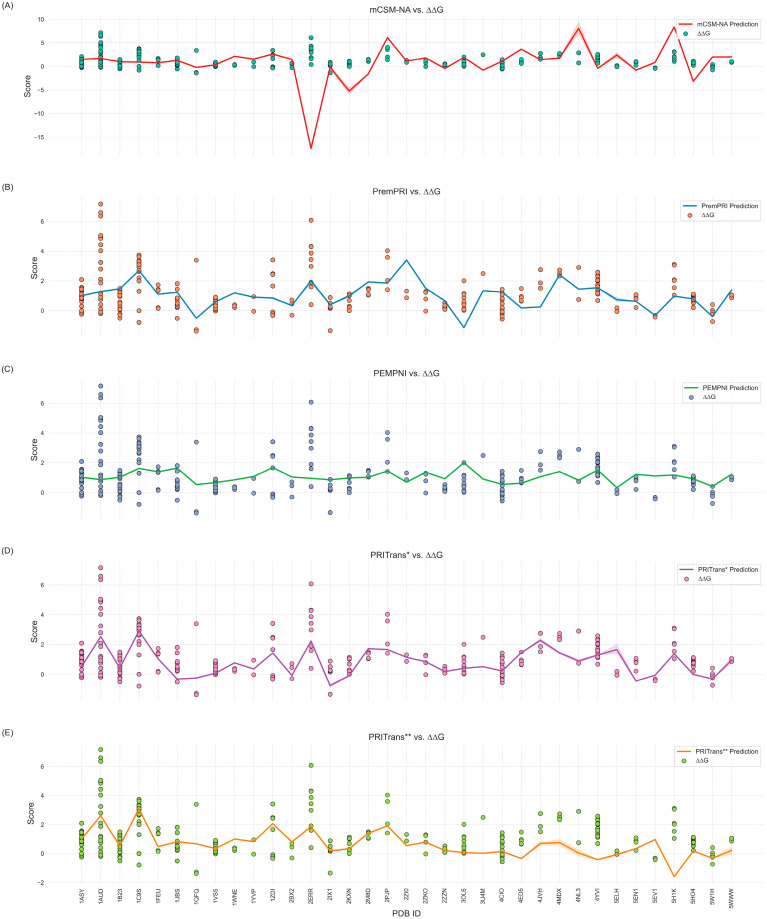
Analysis of prediction results for S79 mutation data using different methods. (**A**–**E**) present predicted versus experimental ΔΔG values for mCSM-NA, PremPRI, PEMPNI, PRITrans*, and PRITrans**, respectively, with each line representing the average predicted values for multiple mutations of each PDB_ID.

To assess the performance of PRITrans, we compared it with three existing methods: PEMPNI [15], PremPRI [29], mCSM-NA [31], which were designed to predict the impact of mutations on protein–RNA interactions. PEMPNI [15] combines energy-based and non-energy-based models to calculate changes in binding affinity, PremPRI [29] uses multiple linear regression, and mCSM-NA [31] employs graph-based signatures. We submitted S79 mutation data to the webservers of these methods and calculated their prediction performance. Results are shown in Table S1 and Figure 3 and Figure 4.

The submission of S79 mutation data to the mCSM-NA webserver yielded a PCC of 0.055 and an RMSE of 4.184 kcal·mol^−1^. After excluding 15 mutation data outliers with squared errors above 12, the PCC improved to 0.384, and the RMSE reduced to 1.486 kcal·mol^−1^. For PremPRI, 13 out of 79 mutations lacked predictions, resulting in a PCC of 0.417 and RMSE of 1.356 kcal·mol^−1^ after exclusion. Including experimental ΔΔG for missing predictions increased the PCC to 0.586 and decreased the RMSE to 1.240 kcal·mol^−1^. Similarly, PEMPNI’s results initially excluded four missing predictions, achieving a PCC of 0.329 and an RMSE of 1.493 kcal·mol^−1^. With the experimental values included, PEMPNI reached a PCC of 0.346 and an RMSE of 1.455 kcal·mol^−1^.

Figure 4A–E illustrates a comparison between predicted and experimental ΔΔG values across different models for the S79 missense mutations data. These figures provide insights into the predictive accuracy and consistency of each method. Figure 4A (mCSM-NA) exhibits substantial variability and notable deviations from the experimental values, particularly for PDB_IDs 2ERR and 1C9S. This suggests a lack of stability in mCSM-NA’s predictions, especially for challenging cases. Figure 4B (PremPRI) shows closer alignment with experimental data compared to mCSM-NA; however, discrepancies remain, particularly for PDB_IDs 1AUD and 3OL6, indicating limitations in its regression-based approach when dealing with complex interactions. Figure 4C (PEMPNI) offers relatively consistent predictions but has marked deviations for PDB_IDs 1AUD and 1C9S, suggesting that while PEMPNI’s energy-based methods capture general trends, they may struggle with specific structural contexts. Figure 4D,E (PRITrans and PRITrans*) shows that PRITrans* displays a strong alignment with experimental data, demonstrating its ability to accurately predict ΔΔG changes. PRITrans extends this capability, showing improved generalization and stability across a broader range of PDB_IDs, including more complex reverse mutations.

Overall, PRITrans* and PRITrans** outperform mCSM-NA, PremPRI, and PEMPNI in predicting the effects of missense mutations on protein–RNA interactions. This enhanced performance is due to the integration of ESM-2 and ProtTrans embeddings with advanced deep learning architectures, such as Transformers and multiscale convolution modules. These techniques allow PRITrans to effectively capture complex mutation patterns, leading to superior predictive accuracy and robustness.

### 2.4. Case Study

Table 5 presents the prediction results for the S79 mutation dataset using PRITrans*, PRITrans**, PEMPNI, PremPRI, and mCSM-NA. Across both high and low ΔΔG mutations, PRITrans* and PRITrans** consistently delivered more accurate predictions compared to the other methods, particularly mCSM-NA. For mutations with high ΔΔG values, the PRITrans models demonstrated superior predictive accuracy. Specifically, for the Q53E mutation (PDB ID: 1AUD, ΔΔG = 6.60 kcal/mol), PRITrans* and PRITrans** predicted values of 3.94 kcal/mol and 5.01 kcal/mol, respectively, which are significantly closer to the experimental result compared to PEMPNI’s 0.781 kcal/mol, PremPRI’s 1.47 kcal/mol, and mCSM-NA’s 2.359 kcal/mol. Similarly, for the G52A mutation (PDB ID: 1AUD, ΔΔG = 3.25 kcal/mol), PRITrans* predicted 3.62 kcal/mol and PRITrans** predicted 3.07 kcal/mol, outperforming PEMPNI (0.985 kcal/mol), PremPRI (0.84 kcal/mol), and mCSM-NA (−0.543 kcal/mol).

Moreover, for mutations with low ΔΔG values, PRITrans exhibited superior performance. For the R181A mutation (PDB ID: 2ZZN, ΔΔG = 0.15 kcal/mol), PRITrans* and PRITrans** predicted 0.00 kcal/mol and 0.17 kcal/mol, respectively, closely matching the experimental value. Likewise, for the K104M mutation (PDB ID: 4CIO, ΔΔG = 0.09 kcal/mol), PRITrans* and PRITrans** achieved predictions of 0.27 kcal/mol and 0.34 kcal/mol, demonstrating higher accuracy than PEMPNI (0.397 kcal/mol), PremPRI (1.30 kcal/mol), and mCSM-NA (2.358 kcal/mol).

**Figure 5:**
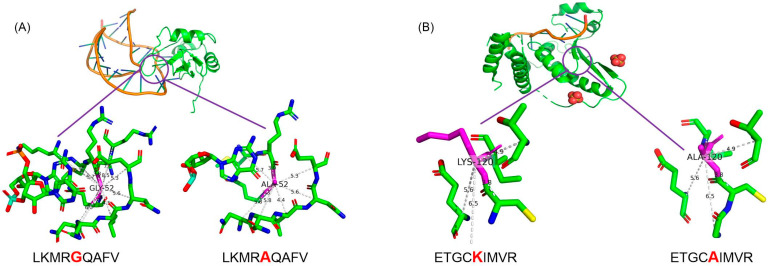
Structural impact of missense mutations on protein-RNA interaction sites. (**A**) shows the interaction site with a mutation (in PDB_ID: 1AUD) from G to A at position 52. (**B**) illustrates the interaction site with a mutation (in PDB_ID: 4JVH) from K to A at position 120.

Figure 5A illustrates the structural alteration in 1AUD following the substitution of glycine (G) with alanine (A) at position 52. Glycine’s small side chain contributes high spatial flexibility to the protein structure; in contrast, alanine, with its slightly bulkier yet non-polar methyl side chain, introduces a limited degree of steric hindrance without altering the electrostatic profile. Consequently, this substitution diminishes spatial flexibility while preserving the neutral charge, which may result in subtle adjustments to local conformation. Figure 5B illustrates changes in 4JVH, where lysine (K) at position 120 is replaced by alanine (A). Lysine, with its long, positively charged side chain, plays a key role in interacting with RNA through electrostatic forces. The replacement with the smaller, neutral alanine significantly weakens these interactions, reducing electrostatic attraction and binding affinity, thereby modifying the local interaction network.

This analysis underscores the importance of side chain chemistry in protein–RNA interactions. The G-to-A mutation increases electrostatic forces and steric effects, while the K-to-A mutation weakens electrostatic interactions. These changes demonstrate how variations in local interaction strength can impact the stability of the interaction interface and potentially alter the overall functionality of the protein–RNA complex.

## 3. Materials and Methods

### 3.1. Reconstruction of Benchmark Datasets

In this study, we systematically collected data regarding missense mutations’ effects on protein–RNA interactions from several published literatures, including PremPRI [29], mCSM-NA [31], PEMPNI [15], and prabhot [36]. To ensure the dataset’s accuracy and reliability, we removed duplicate mutations, resolved inconsistencies where the same mutation had conflicting ∆∆G values across sources, and excluded cases with incorrect amino acid sequences in wild-type proteins (e.g., PDB_ID: 2ZZM). After this stringent filtering, we obtained a high-quality dataset of 394 mutations from 78 protein–RNA complexes, designated S394. Recognizing the dataset’s natural bias, where only 41 mutations exhibited ΔΔG < 0 (∆∆G = ∆G_mutant_ − ∆G_wild-type_, where ∆G_wild-type_ and ∆G_mutant_ represent the binding free energy of the wild-type and mutant protein-RNA complex, respectively), we applied thermodynamic reversibility to each mutation. This ensured that ΔΔG (wild-type → mutant) equaled –ΔΔG (mutant → wild-type). As a result, we generated 394 reverse mutations, expanding the dataset to a total of 788 mutations, referred to as S788.

For the purpose of training and validation, we randomly partitioned the S394 dataset into an 80% training set (S315) and a 20% independent test set (S79). To further refine our analysis, we applied a similar division to the forward and reverse mutation dataset S788, producing subsets S630 and S158, respectively. Detailed statistical details regarding these mutation datasets are provided in Table 6, and a summary of the names and specific uses of the various sub-datasets are provided in Table S2.

### 3.2. Feature Representation

Extracting numerical features from protein sequences is essential for developing deep learning models that assess the impacts of missense mutations on protein–RNA interactions [45,46,47,48]. Recent advancements in protein language models, such as ESM-2 [43] and ProtTrans [44], have significantly improved the accuracy of protein property predictions by incorporating sequence conservation [41,49]. In this study, we focused on truncated mutant sequences centered at the mutation site and determined that an optimal length of 181 residues yielded the best results after testing various sequence lengths (see Text S1 and Tables S3 and S4 for details). We then used ESM-2 [43] and ProtTrans [44] to generate embeddings with 181 × 1280 and 181 × 1024 dimensions, facilitating characterization of mutations.

### 3.3. Architecture of PRITrans

**Figure 6:**
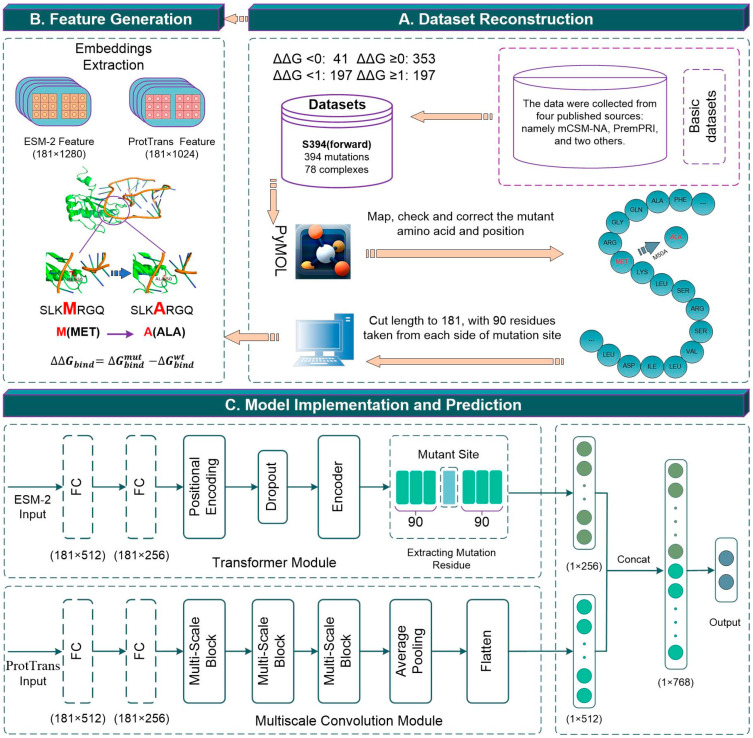
Workflow of PRITrans. (**A**) Dataset reconstruction. (**B**) Feature generation. (**C**) Model implementation and prediction. Note: as illustrated in the “Extracting Mutation Residue” part of (**C**), the central light blue region represents the mutant site, whereas the adjacent green regions depict the 90 amino acid residues positioned upstream and downstream of the mutant site, respectively.

The proposed PRITrans framework, shown in Figure 6, consists of three key components: (1) Construction of a benchmark dataset (Figure 6A). (2) Feature extraction (Figure 6B), where two feature matrices are generated: ESM-2 (181 × 1280) and ProtTrans (181 × 1024). (3) Model implementation and prediction (Figure 6C), which processes ESM-2 and ProtTrans features separately. ESM-2 embeddings are first passed through a transformer module, producing a 181 × 256 feature matrix that captures the mutation site’s 256-dimensional features. Simultaneously, ProtTrans embeddings are reduced from 1024 to 256 dimensions via two fully connected layers, followed by a multiscale convolution module, resulting in a 512-dimensional feature set (additional details are in Figure S4). The features from both models are then integrated, flattened, and concatenated. The combined feature set is processed through three nonlinear fully connected layers with 256, 128, and 1 neuron(s), respectively, and a dropout rate of 0.3. The final layer predicts the ∆∆G value, indicating whether a mutation significantly impacts binding affinity.

In PRITrans, we optimized key hyperparameters such as the learning rate (“lr”), parameters within the Encoder module, the dropout rate (“rate”) applied to the final fully connected layer, and the L2 regularization parameter (“l2_reg”). Through comparative experiments, we determined that the optimal configuration for this task includes a learning rate of 1 × 10^−4^, a dropout rate of 0.3, and an L2 regularization of 1 × 10^−3^.

#### 3.3.1. Encoder Module

The Transformer module comprises two non-linear fully connected layers, positional encoding, and an encoder. Positional encoding enriches protein embeddings by integrating positional and semantic information. The encoder, as shown in Figure S4A, transforms input sequences into high-dimensional representations using two identical layers. A multi-head attention layer first processes the input matrix with distinct attention heads, capturing diverse features through scaled dot-product attention, which utilizes query (Q), key (K), and value (V) inputs. Q computes attention weights, while K and V generate weighted representations, emphasizing relevant contextual information. The outputs from the attention heads are concatenated and linearly transformed. A feedforward network then applies two linear transformations to introduce non-linear features. Layer normalization accelerates convergence, while dropout layers and residual connections prevent gradient vanishing and overfitting, improving robustness and generalization.

In the encoder module, the parameter num_layers determines the number of encoder layers. Increasing this number deepens the model, thereby enhancing its representational capacity. The parameter d_model defines the feature dimension, setting the size of the feature representations at each encoder position and influencing the model’s expressiveness. Additionally, num_heads specifies the number of attention heads in the self-attention layer, indicating how many subspaces are used for parallel attention computations, which enhances the model’s ability to learn diverse features.

As depicted in Figure S5, we explored the effects of varying encoder layer counts and attention head numbers on predictive performance with d_model fixed at 512. The results show that using two encoder layers and four attention heads yields the best performance on both cross-validation and independent test sets compared to other configurations. Therefore, we selected 2, 512, and 4 as the final values for num_layers, d_model, and num_heads, respectively.

#### 3.3.2. Multiscale Convolution Module

The multiscale convolution module consists of three blocks (Figure S4B), each with three sub-layers designed to leverage convolutional kernels of different sizes (3 × 3, 5 × 5, 7 × 7) for feature extraction. Each sub-layer includes a convolutional, activation, batch normalization, and max pooling layer. The outputs are combined and passed to the next block. ReLU activation mitigates overfitting, while batch normalization accelerates training and improves accuracy [50]. Max pooling captures essential features by selecting the maximum values from the previous layer, reducing computational load [51].

### 3.4. Evaluation Metrics and Cross-Validation Strategies

To rigorously and systematically [52] assess PRITrans’s performance and compare it to existing predictors, we used three regression metrics: PCC, RMSE, and MAE. PCC measures the correlation between predicted and actual values, with values closer to 1 indicating a stronger linear relationship. RMSE and MAE assess prediction errors, where lower values represent better performance (definitions in Text S2).

We used three cross-validation strategies: CV1, CV2, and CV3. In CV1, the dataset was split into 90% training data (10% for validation) and 10% test data. In CV2, 80% of the data was used for training (10% for validation), with 20% reserved for testing. Both CV1 and CV2 were repeated 20 times, and the metrics were averaged to ensure robustness. CV3 employed ten-fold cross-validation, averaging metrics over the folds for a more comprehensive evaluation.

## 4. Conclusions

We have developed PRITrans, a deep learning model based on the Transformer architecture, to predict the effects of missense mutations on protein-RNA interactions. The primary innovation of PRITrans lies in its utilization of protein language model embeddings, which effectively capture and represent critical sequence features without the need for handcrafted inputs, thereby demonstrating strong adaptability. Moreover, PRITrans’s architecture seamlessly integrates these complex features, enabling more comprehensive and detailed analyses than traditional models. While embeddings alone may limit biological interpretability when used in isolation, PRITrans overcomes this limitation by incorporating feature fusion and contextual integration, which enhances both predictive accuracy and biological insight. This methodology has exhibited superior performance across diverse datasets, underscoring its methodological strengths.

Future research will focus on refining PRITrans for greater precision and expanding its applicability to key areas in bioinformatics, such as protein–protein interactions, protein–DNA interactions, protein–lipid complex modeling, and antibody–antigen interactions. These fields are essential for elucidating complex biological systems and understanding disease mechanisms, where accurate modeling of molecular interactions is vital. By leveraging its advanced Transformer architecture and self-attention mechanisms, PRITrans is expected to achieve significant improvements in predictive power and flexibility across these challenges. Furthermore, through modeling complex interfaces, analyzing sequence specificity, and evaluating the impacts of mutations on structure and function, PRITrans has the potential to significantly advance both research and practical applications.

To further enhance biological interpretability and generalization, future efforts will concentrate on integrating additional protein structural features and incorporating a wider range of biological datasets. We believe that PRITrans holds substantial potential to advance biomedical research, facilitate scientific discovery, and enhance practical applications.

## Figures and Tables

**Table 1:** The performance of PRITrans on S315 and S630 datasets using CV1, CV2, and CV3.

Dataset	Cross-Validation Strategies	PCC	RMSE (kcal·mol^−1^)	MAE (kcal·mol^−1^)
S315 (Forward)	CV1	0.776 ± 0.048	0.768 ± 0.094	0.557 ± 0.052
	CV2	0.743 ± 0.034	0.744 ± 0.054	0.538 ± 0.039
	CV3	0.581 ± 0.184	1.071 ± 0.307	0.808 ± 0.226
S630 (Forward + Reverse)	CV1	0.729 ± 0.047	1.250 ± 0.094	0.794 ± 0.060
	CV2	0.728 ± 0.017	1.197 ± 0.038	0.850 ± 0.033
	CV3	0.741 ± 0.065	1.168 ± 0.205	0.809 ± 0.112

Note: CV1 and CV2 were each repeated 20 times, with the average values taken as the final result.

**Table 2:** Performance of PRITrans with different encoder configurations using CV3 on S630.

PRITrans with Different Encoder Module	PCC	RMSE (kcal·mol^−1^)	MAE (kcal·mol^−1^)
ESM-2_p_ + PT_p_	0.610	1.372	0.983
ESM-2_Ep_ + PT_p_	0.641	1.339	0.943
ESM-2_p_ + PT_Ep_	0.614	1.391	0.983
ESM-2_Ep_ + PT_Ep_	0.670	1.315	0.918

**Table 3:** Performance of PRITrans with different convolution modules using CV3 on S360.

PRITrans with Different Convolution Module	PCC	RMSE (kcal·mol^−1^)	MAE (kcal·mol^−1^)
ESM-2_m_ + PT_p_	0.597	1.435	1.028
ESM-2_p_ + PT_m_	0.684	1.284	0.902
ESM-2_m_ + PT_m_	0.750	1.207	0.861

**Table 4:** Performance of PRITrans with combined modules using CV3 on S630.

PRITrans with Different Modules	PCC	RMSE (kcal·mol^−1^)	MAE (kcal·mol^−1^)
ESM-2_Ep_	0.674	1.328	0.841
PT_m_	0.601	1.495	1.117
ESM-2_Ep_ + PT_m_	0.741	1.168	0.809

**Table 5:** Prediction results of four methods on the S79 mutation data.

PDB_ID	Chain	Mutation	∆∆G	PRITrans*_pred	PRITrans**_pred	PEMPNI_pred	PremPRI_pred	mCSM-NA_pred
1AUD	A	G52A	3.25	3.62	3.07	0.985	0.84	−0.543
1AUD	A	Q53A	4.85	4.79	3.44	1.147	1.06	2.166
1AUD	A	Q53E	6.6	3.94	5.01	0.781	1.47	2.359
1B23	P	K90A	0.57	0.15	0.22	0.920	1.51	1.920
1B23	P	N64A	−0.51	−0.05	0.26	1.031	1.26	0.562
2M8D	B	K138A	1.43	1.72	1.40	1.020	1.93	−1.574
2ZZN	A	R181A	0.15	0.00	0.17	1.010	0.21	0.681
2ZZN	A	N265Q	0.08	0.35	0.23	0.837	1.10	−1.576
4CIO	A	K104M	0.09	0.27	0.34	0.397	1.30	2.358
4JVH	A	K120A	1.87	1.69	1.29	0.877	0.23	2.094

Note: PRITrans* refers to PRITrans trained on the forward data using CV3. PRITrans** indicates PRITrans trained on the entire dataset using CV3.

**Table 6:** Detailed statistical information of benchmark datasets.

Dataset	Mutation Type	Complex Count	Mutation Count	∆∆G < 0	∆∆G ≥ 0	∆∆G < 1	∆∆G ≥ 1
S394	Forward	78	394	41	353	197	197
S315	Forward for training	68	315	31	284	161	154
S79	Forward for independent test	35	79	10	69	36	43
S630	Forward and reverse for training	68	630	309	321	/	/
S158	Forward and reverse for independent test	35	158	79	79	/	/

Note: S315 (80% of S394) + S79 = S394, S630 (S315 forward + S315 reverse) + S158 (S79 forward + S79 reverse) = S788. ∆∆G > 0 indicates the mutation decreases protein-RNA binding affinity.

## Data Availability

The reconstructed benchmark datasets and codes are available at https://github.com/cuifengLI/PRITrans (accessed on 14 November 2024).

## Supplementary Materials

The following supporting information can be downloaded at: https://www.mdpi.com/article/10.3390/ijms252212348/s1.
