# Supplementary material for: PRITrans: A Transformer-Based Approach for the Prediction of the Effects of Missense Mutation on Protein–RNA Interactions
Source: Int J Mol Sci. 2024 Nov 17;25(22):12348. doi: 10.3390/ijms252212348 (PMC11594650; doi:10.3390/ijms252212348)
Supplement: Supplementary file 1 [file ijms-25-12348-s001.zip › ijms-3275662-supplementary.pdf]

Supplementary Material

PRITrans: A Transformer-Based Approach for the Prediction of the Effects of Missense Mutation on Protein–RNA Interactions

Fang Ge [1,†], Cui-Feng Li [2,†], Chao-Ming Zhang [2], Ming Zhang [2] and Dong-Jun Yu [3,*]

1 State Key Laboratory of Organic Electronics and Information Displays & Institute of Advanced Materials (IAM), Nanjing University of Posts & Telecommunications, 9 Wenyuan, Nanjing 210023, China; gfang0616@njupt.edu.cn

2 School of Computer, Jiangsu University of Science and Technology, 666 Changhui Road, Zhenjiang 212100, China; 231210701110@stu.just.edu.cn (C.-F.L.); 221210701126@stu.just.edu.cn (C.-M.Z.); zhangming@just.edu.cn (M.Z.)

3 School of Computer Science and Engineering, Nanjing University of Science and Technology, 200 Xiaolingwei, Nanjing 210094, China

* Correspondence: njyudj@njust.edu.cn

† These authors contributed equally to this work.

Supplementary Texts

Supplementary Text S1

Text S1. Determining optimal protein sub_sequence lengths for feature extraction.

To identify the optimal sub-sequence length for protein feature extraction, we tested various window sizes (W = 50, 60, 70, 80, 90, 100) centered on mutation sites. For W = 90, a sub-sequence of 181 residues is generated, encompassing 90 residues upstream and downstream of the mutation site. For sequences shorter than 90 residues, padding with 'X' was applied. Extracted features from pre-trained ESM and ProtTrans models were then fed into the PRITrans Transformer module, and performance was evaluated using the CV3 cross-validation strategy on benchmark datasets S315 and S630. As shown in Tables S2 and S3, the model performed worst at W = 50, with PCCs of 0.539 and 0.647 and RMSEs of 1.153 and 1.354 on S315 and S630, respectively. Performance improved with W = 80, achieving PCCs of 0.578 and 0.727 and RMSEs of 1.080 and 1.208. The best results were obtained with W = 90, yielding PCCs of 0.581 and 0.741 and RMSEs of 1.071 and 1.168 on S315 and S630, representing improvements of 0.003 and 0.014 in PCC compared to W = 80. Performance metrics consistently improved as W increased from 50 to 90, but declined when W reached 100. Therefore, W = 90 was selected as the optimal sub-sequence length for feature extraction based on these findings.

Supplementary Text S2

Text S2. Performance evaluation metrics.

The model's performance is assessed using three key metrics: Pearson Correlation Coefficient (PCC), Root Mean Square Error (RMSE), and Mean Absolute Error (MAE), defined as follows:

$$PCC = \frac{n\sum x_i y_i - \sum x_i \sum y_i}{\sqrt{n\sum x_i^2 - \left(\sum x_i\right)^2}\sqrt{n\sum y_i^2 - \left(\sum y_i\right)^2}} \tag{S1}$$

$$RMSE = \sqrt{\frac{1}{n}\sum_{i=1}^{n}\left(y_i - x_i\right)^2} \tag{S2}$$

$$MAE = \sqrt{\frac{1}{n}\sum_{i=1}^{n}\left|y_i - x_i\right|^2} \tag{S3}$$

In Equations (S1) to (S3), i denotes the ith mutation, n is the total number of mutations, x represents the experimental ΔΔG value, and y represents the predicted ΔΔG value. PCC measures the linear correlation between experimental and predicted values, indicating the strength of their relationship. RMSE captures the average size of prediction errors, with a greater emphasis on larger deviations. MAE measures the average absolute differences between experimental and predicted values, providing a straightforward assessment of prediction accuracy.

Supplementary Figures

Supplementary Figure S1

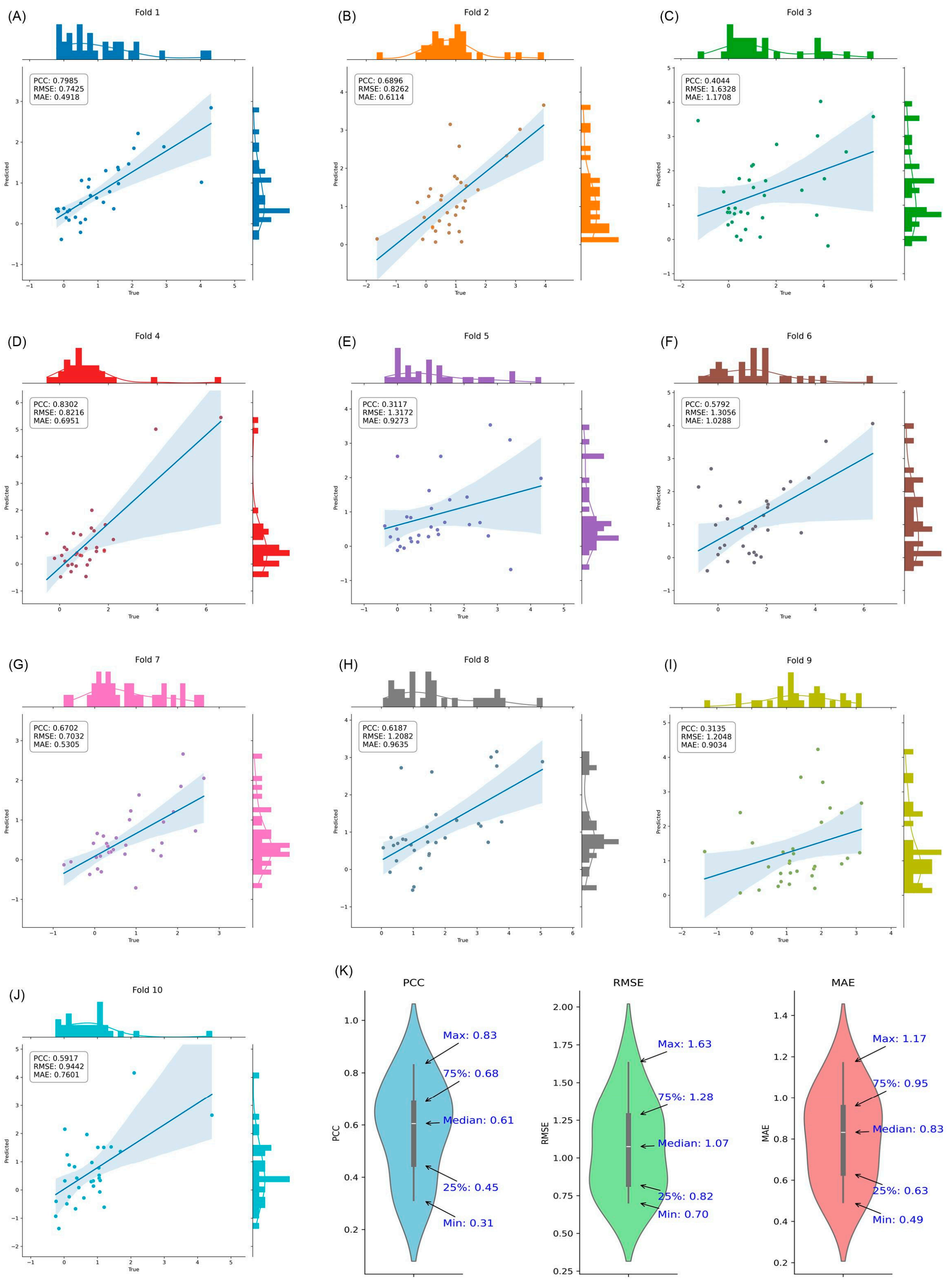

Figure S1. Performance of PRITrans on the S315 dataset using CV3. **(A)** to **(J)** present the scatter plots for Fold_1 to Fold_10, illustrating the relationship between actual and predicted values. **(K)** provides the violin plots for PCC, RMSE, and MAE across Fold_1 to Fold_10.

Supplementary Figure S2

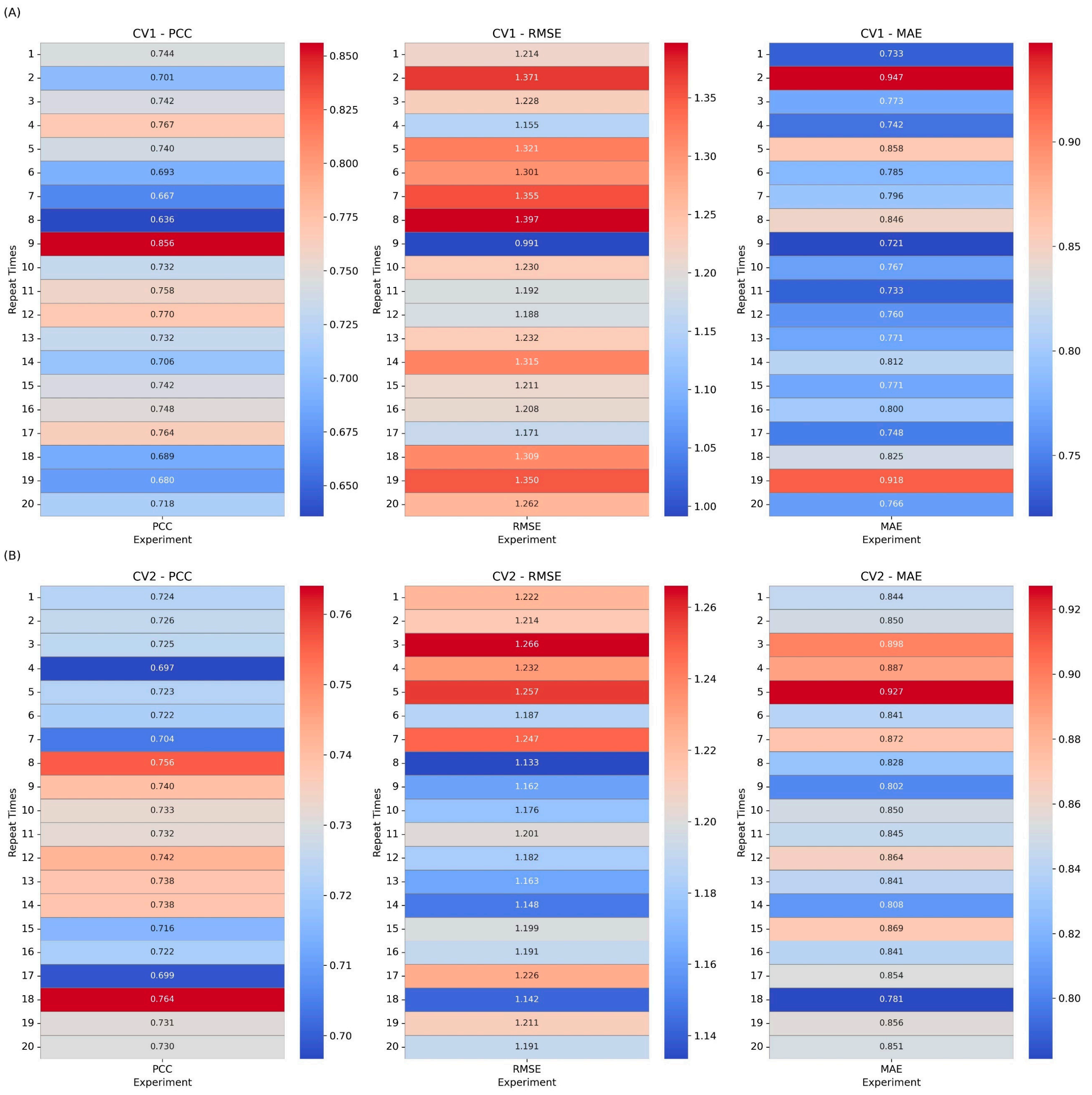

Figure S2. Heatmap of prediction results using CV1 and CV2 on S630 (repeated 20 times). **(A)-(B)** Heatmap for CV1 and CV2.

Supplementary Figure S3

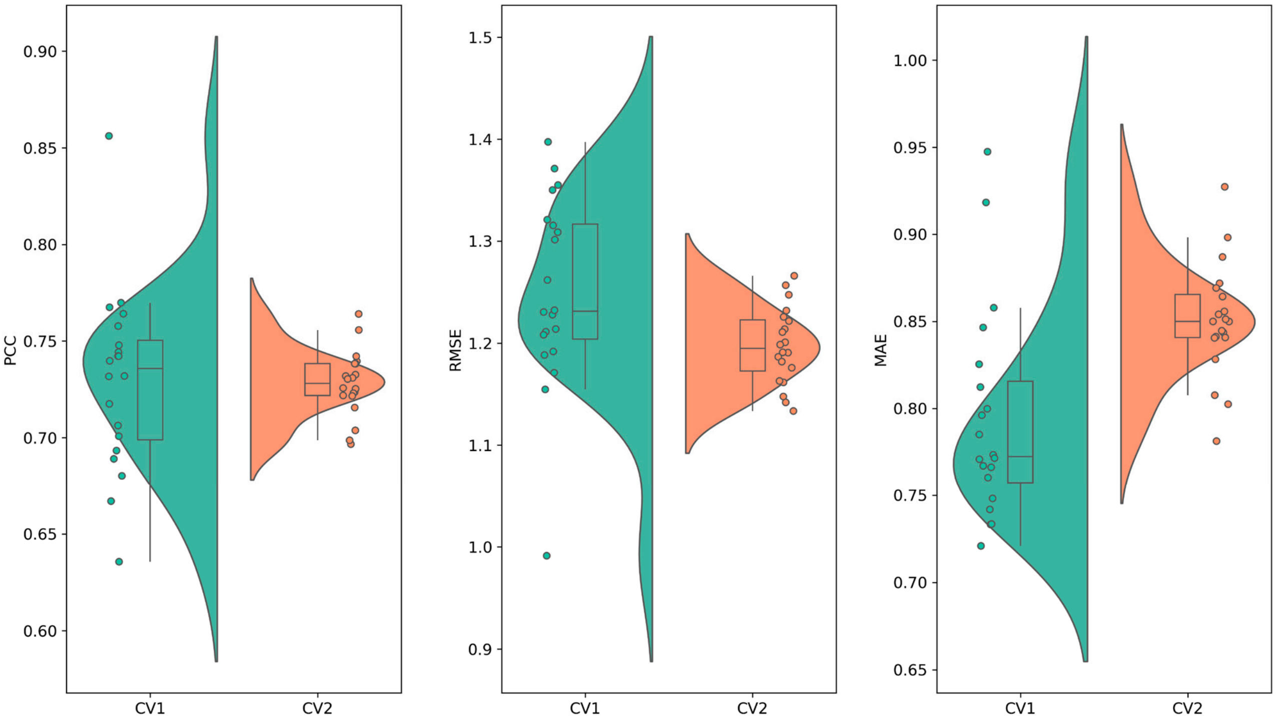

Figure S3. Comparison metrics of CV1 and CV2 on the S630 dataset. **(A)-(C)** show the violin plots for PCC, RMSE, and MAE.

Supplementary Figure S4

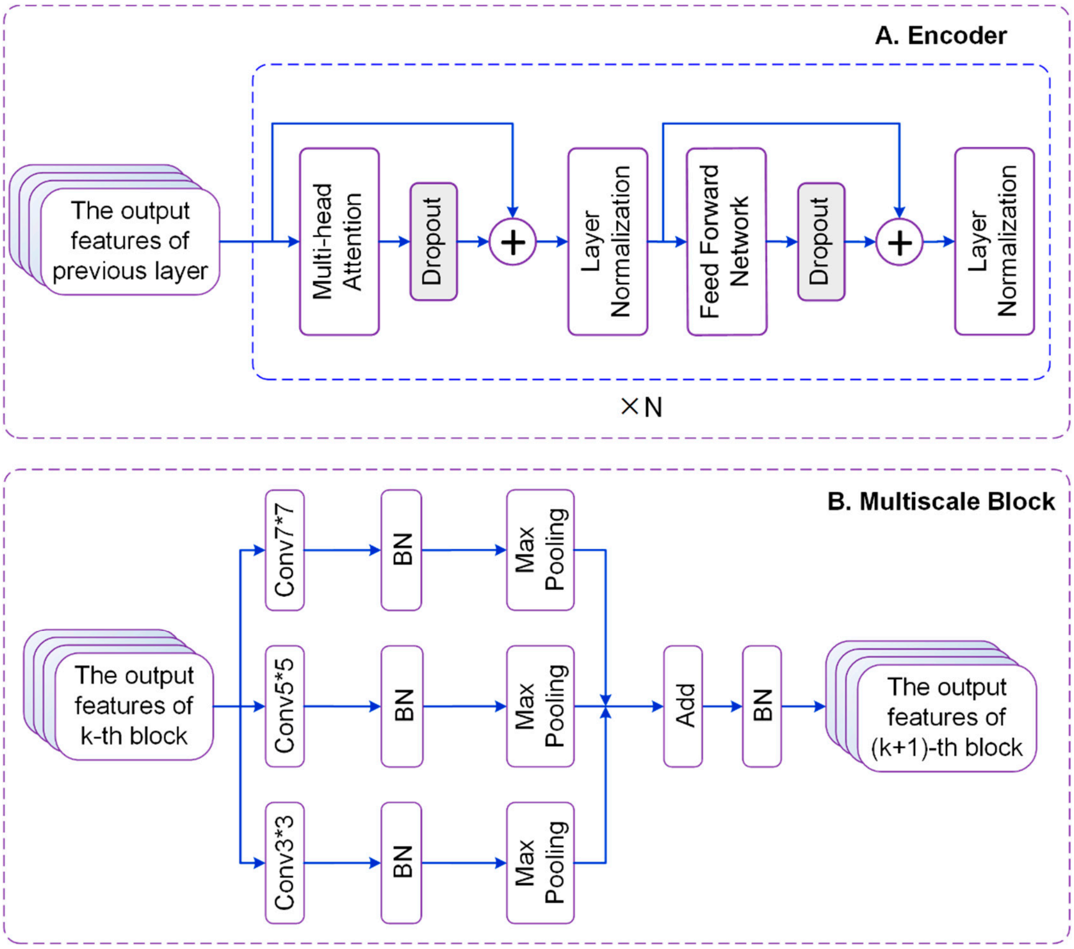

Figure S4. Detailed architecture of the Encoder module and Multiscale block module.

Supplementary Figure S5

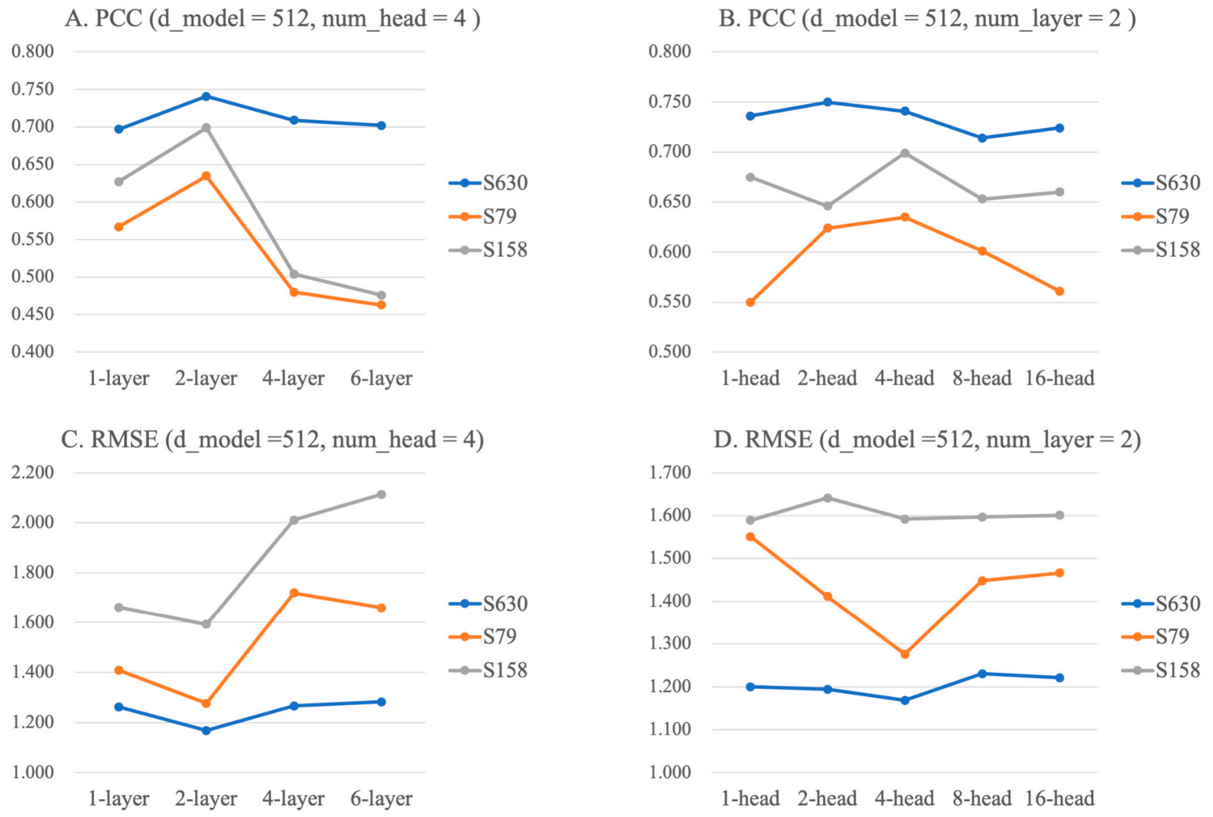

Figure S5. Model performance with different numbers of Encoder layers and multiheads when d_model is 512. (A)-(D) show the PCC and RMSE when num_head is 4 and num_layer is 2 for S630, S79, S158, respectively.

Supplementary Tables

Supplementary Table S1

Table S1. Performance comparison of PRITrans and existing predictors using S79 mutation data.

Method	Data name	PCC	RMSE (kcal·mol^{-1})	MAE (kcal·mol^{-1})
PRITrans*	S79	0.751	1.068	0.787
PRITrans***	S158	0.699	1.592	1.126
PRITrans**	S79	0.635	1.276	0.934
mCSM-NA	S79	0.055	4.184	2.360
PremPRI*	S79	0.586	1.240	0.784
PEMPNI*	S79	0.346	1.455	0.911
mCSM-NA*	S64	0.384	1.486	1.205
PremPRI	S66	0.417	1.356	0.938
PEMPNI	S75	0.329	1.493	0.960

Note: PRITrans*, trained on forward data using CV3. PRITrans**, trained on the entire dataset using CV3. PRITrans***, trained on the entire dataset using CV3 and evaluated on the S158 dataset, including reverse mutations. Additionally, PremPRI*, missing predictions for PDB_IDs 1C9S (10), 4MDX (2), and 5EV1 (1) were substituted with experimental ΔΔG values. PEMPNI*, Missing predictions for PDB_IDs 1VS5 (2), 3OL6 (1), and 5W1H (1) were replaced with experimental ΔΔG values. mCSM-NA*, excludes the 15 data points with the highest squared errors between predictions and experimental ΔΔG values.

Supplementary Table S2

Table S2. Detailed dataset information of PRITrans, PremPRI, mCSM-NA, PEMPNI, and Prabhot used for cross-validation.

<table>
<tr><th colspan="2">Dataset</th><th>Mutation Count</th><th>Complex Count</th><th>Description</th></tr>
<tr><td colspan="2">S394</td><td>394</td><td>78</td><td>constructed benchmark dataset of PRITrans (only containing forward mutations)</td></tr>
<tr><td colspan="2">S788</td><td>788</td><td>78</td><td>constructed benchmark dataset of PRITrans (containing forward + reverse mutations)</td></tr>
<tr><td colspan="2">S509</td><td>509(233)</td><td>100(47)</td><td>training set of PEMPNI[1], including mutations from both protein-DNA and protein-RNA complexes; the numbers in parentheses indicate the number of mutations from protein-RNA complexes.</td></tr>
<tr><td colspan="2">S248</td><td>248</td><td>50</td><td>training set of PremPRI[2]</td></tr>
<tr><td colspan="2">S264</td><td>264(67*)</td><td>33(5)</td><td>training set of mCSM-NA[3], including mutations from both protein-DNA and protein-RNA complexes; the numbers in parentheses indicate the mutations from protein-RNA complexes.</td></tr>
<tr><td colspan="2">S151</td><td>151</td><td>32</td><td>benchmark dataset of Prabhot[4]</td></tr>
<tr><td colspan="2">S58</td><td>58</td><td>15</td><td>independent dataset of Prabhot[4]</td></tr>
<tr><td colspan="2">S79/MPR79</td><td>79</td><td>14</td><td>independent dataset of mCSM-NA[3] and PEMPNI[1]</td></tr>
<tr><td rowspan="6">S394</td><td>S212</td><td>212</td><td>47</td><td>overlap mutation count between S394 and S248</td></tr>
<tr><td>S52</td><td>52</td><td>12</td><td>mutations from S233 are added into S212</td></tr>
<tr><td>S48</td><td>48</td><td>3</td><td>mutations from S67 are added into S212</td></tr>
<tr><td>S26</td><td>26</td><td>6</td><td>mutations from S151 are added into S212</td></tr>
<tr><td>S16</td><td>16</td><td>5</td><td>mutations from S58 are added into S212</td></tr>
<tr><td>S40</td><td>40</td><td>5</td><td>mutations from S79 are added into S212</td></tr>
</table>

Note: in mCSM-NA[3], the dataset values are the inverse of those in the baseline dataset S248. Therefore, we use the baseline dataset S248 as the primary reference and take the inverse values for the extended dataset in mCSM-NA.

Supplementary Table S3

Table S3. Comparison of experimental results using different W values for cutting protein sub-sequences on the forward mutation data using the CV3 strategy.

Evaluation Metrics	W=50	W=60	W=70	W=80	W=90	W=100
PCC	0.539	0.562	0.571	0.578	0.581	0.550
RMSE ($kcal \cdot mol^{-1}$)	1.153	1.114	1.097	1.080	1.071	1.140
MAE ($kcal \cdot mol^{-1}$)	0.855	0.827	0.818	0.826	0.808	0.849

Supplementary Table S4

Table S4. Comparison of experimental results using different W values for cutting protein sub-sequences on the forward and reverse mutation data using the CV3 strategy.

Evaluation Metrics	W=50	W=60	W=70	W=80	W=90	W=100
PCC	0.647	0.702	0.710	0.727	0.741	0.724
RMSE ($kcal \cdot mol^{-1}$)	1.354	1.249	1.248	1.208	1.168	1.239
MAE ($kcal \cdot mol^{-1}$)	0.962	0.899	0.861	0.850	0.809	0.851

References

1. Jiang, Y.; Liu, H.-F.; Liu, R., Systematic comparison and prediction of the effects of missense mutations on protein-DNA and protein-RNA interactions. *PLoS Computational Biology* **2021,** 17, (4), e1008951.
2. Zhang, N.; Lu, H.; Chen, Y.; Zhu, Z.; Yang, Q.; Wang, S.; Li, M., PremPRI: Predicting the effects of missense mutations on protein–RNA interactions. *International journal of molecular sciences* **2020,** 21, (15), 5560.
3. Pires, D. E.; Ascher, D. B., mCSM–NA: predicting the effects of mutations on protein–nucleic acids interactions. *Nucleic acids research* **2017,** 45, (W1), W241-W246.
4. Pan, Y.; Wang, Z.; Zhan, W.; Deng, L., Computational identification of binding energy hot spots in protein–RNA complexes using an ensemble approach. *Bioinformatics* **2018,** 34, (9), 1473-1480.
